# Prolactin levels in functional hypothalamic amenorrhea: a retrospective case–control study

**DOI:** 10.1007/s00404-023-07277-1

**Published:** 2023-11-14

**Authors:** Clara Selzer, Johannes Ott, Didier Dewailly, Rodrig Marculescu, Johanna Steininger, Marlene Hager

**Affiliations:** 1 Clinical Division of Gynecological Endocrinology and Reproductive Medicine, Department of Obstetrics and Gynecology, Medical University of Vienna, Spitalgasse 23, 1090 Vienna, Austria; 2 Faculty of Medicine Henri Warembourg, University of Lille, Lille Cedex, France; 3 Department of Laboratory Medicine, Medical University of Vienna, Spitalgasse 23, 1090 Vienna, Austria

**Keywords:** Hypogonadotropic hypogonadism, Prolactin, Stress, Polycystic ovary syndrome

## Abstract

**Purpose:**

Functional hypothalamic amenorrhea (FHA) is due to hypothalamic dysregulation. Literature lacks data about prolactin in FHA women, although both prolactin levels and FHA are associated with stress. Moreover, polycystic ovarian morphology is common in FHA and there is an association between FHA and polycystic ovary syndrome. Thus, the aim of this study was to assess prolactin levels in FHA patients and controls with a special focus on factors influencing prolactin levels, that could be considered as “sensors” of the hypothalamic–pituitary dysregulation.

**Methods:**

In a retrospective cohort study, 140 women with clearly defined FHA were compared to 70 healthy, normally ovulating women matched for age. The main outcome parameter was prolactin. Factors associated with prolactin levels > 12 µg/L were tested using a multivariable binary logistic regression model.

**Results:**

The median prolactin level was 11.5 µg/L (interquartile range, IQR 7.5–14.4), which was similar to the control group (median 10.7, IQR 8.3–14.5; *p* = 0.065). Only two women had hyperprolactinemia (prolactin > 25 µg/L; 1.4%). In a multivariable binary logistic regression model eating disorder (odds ratio, OR 0.206; *p* = 0.040), excessive exercise (OR 0.280; *p* = 0.031) and TSH (OR 1.923; *p* = 0.020) were significantly associated with prolactin levels > 12 µg/L.

**Conclusion:**

Women with FHA have similar prolactin levels to healthy age-matched individuals. Eating disorders and excessive exercise where associated with prolactin levels < 12 µg/L, in contrast to TSH.

## What does this study add to the clinical work?

**Table:** 

Even though women with FHA have similar prolactin levels than healthy age-matched individuals, prolactin levels in FHA women could be considered as a “sensor” of the hypothalamic–pituitary dysregulation. It seems that prolactin levels in FHA women are mainly influenced by metabolic causes.	

## Introduction

Functional hypothalamic amenorrhea (FHA) and hyperprolactinemia are common causes of secondary amenorrhea in women of reproductive age amongst other conditions like polycystic ovary syndrome (PCOS) [1]. Women with FHA suffer from chronic anovulation due to a reduced GnRH pulsatility and, therefore, a decrease in LH and FSH pulse frequency, which leads to an impaired folliculogenesis [2–4].

Little is known about prolactin in FHA patients. This is reasonable, since hyperprolactinemia is an exclusion criterion for FHA [5]. However, there are two main reasons to address this lack of evidence. First, prolactin levels could be considered as a “sensor” of the hypothalamic–pituitary dysregulation even when prolactin levels are within the normal range [6]. Notably, it has been claimed that there is a GnRH-stimulated PRL release, which might be mediated by a paracrine FSH effect [7]. Moreover, it has been mentioned that pulsatile release of PRL and LH could be mediated through a common neuroendocrine mechanism [8].

In detail, dopamine exerts effects on prolactin and luteinizing hormone (LH) secretion [9]. Notably, dopamine is involved in central stress responses. Exposure to stress is followed by an increase in the hypothalamic concentration of beta-endorphin, which impairs pulsatile GnRH release and inhibits dopamine secretion of dopamine. Hypothetically, this should cause an increase in prolactin levels [10]. Notably, there are three main causes of FHA according to the Endocrine Society, namely weight loss, and/or vigorous exercise, and/or stress [11]. It has already been reported that excessive stress and exercise can lead to a physiological elevation of prolactin [12]. Physical and psychological stress being triggers of both, FHA and hyperprolactinemia, it seems intuitive that there might be a causal link.

Second, FHA is often associated with polycystic ovarian morphology (PCOM) with a prevalence ranging from 30 to 50%, whereas the prevalence of PCOM in the general population is significantly lower (7–24%). Several studies suggested that women with FHA and PCOM would reveal some metabolic and hormonal patterns which are similar to those found in PCOS [13–15]. Thus, it has been suggested that these patients might have suffered from PCOS before having acquired FHA [14, 15]. Notably, the prevalence of hyperprolactinemia in women with PCOS has been the subject of many previous studies. Literature provides reasonable approaches to explain hyperprolactinemia in women with PCOS: a decrease in dopaminergic tone, relative hyperestrogenemia or an increase in GnRH pulsatility [16, 17]. Moreover, beside its eponymous effect on inducing lactation, prolactin affects metabolism, osmoregulation, immune function, growth, brain and behavior, angiogenesis and many more [12, 18]. Recently, there is evidence that high prolactin levels below and above the conventional hyperprolactinemic threshold (25 µg/L) could have beneficial effects on metabolism, whereas hypoprolactinemia (< 7 µg/L) could be associated with negative metabolic effects like visceral fat dysfunction and insulin resistance [18–20]. In contrast, in case of pathologically high prolactin levels, there are studies that show a higher prevalence of obesity, glucose intolerance and insulin resistance [21]. Given that women with PCOS reveal increased risks for metabolic complications [22], FHA patients with a tendency toward PCOS, i.e. those who reveal PCOM on ultrasound, could also show PCOS-typical alterations in prolactin levels.

Based on all of these considerations and due to the above-mentioned lack of studies about prolactin in FHA patients, the aim of this retrospective cohort study was to evaluate prolactin levels in women with FHA and to compare these to a group of healthy age-matched controls. A special focus was on factors influencing prolactin levels, that could be considered as “sensors” of the hypothalamic–pituitary dysregulation as mentioned above. The FHA subgroups with and without PCOM are of considerably major interest here.

## Methods

### Study population and study design

This retrospective cohort study was conducted at the Clinical Division of Gynecologic Endocrinology and Reproductive Medicine of the Medical University of Vienna, Austria. For the case group, all women with FHA, which were considered eligible and were seen at the department from January 2017 to March 2023, were included. The following inclusion criteria were applied: (i) FHA, defined by strict definition criteria, as previously reported [23]: secondary amenorrhea for at least 6 months; a negative progestogen challenge test; with context of weight loss, insufficient caloric intake, intense physical activity (exercising at least 10 h per week, which included any type of exercise or running at least 30 miles per week) or notion of recent psychological stress (history of emotionally stressful events preceding the onset of amenorrhea included problems within the family, at school, at work or of psychosocial stress, confirmed by a psychologic report). Pregnancy, hypothyroidism, use of any antipsychotic or antidepressive agents, use of dopaminagonists and any organ-related pituitary dysfunction on pituitary MRI had to be excluded. Moreover, a BMI > 30.0 kg/m^2^ was also an exclusion criterion to exclude women with obesity-related FHA. The study was approved by the local ethics committee (IRB number 1019/2023).

For the control group, 70 healthy, normally ovulating controls which had been recruited for previous studies, published [24, 25] or unpublished, were chosen. Controls were matched for age using propensity score matching. Matching for BMI was not possible due to the high rate of women with a low BMI in the FHA group.

### Parameters analyzed

As reported previously [13, 15, 23, 26], the AKIM-software was used for data acquisition. Blood samples were obtained during amenorrhea and were analyzed at the local ISO-certified Department of Laboratory Medicine, General Hospital of Vienna, Vienna, Austria according to ISO 15189 quality standards: estradiol, follicle-stimulating hormone (FSH), LH, prolactin, AMH, testosterone, dehydroepiandrosterone-sulphate (DHEAS), and sex hormone-binding globulin (SHBG) were measured by the corresponding Cobas electrochemiluminescence immunoassays (ECLIA) on Cobas e 602 analyzers (Roche, Mannheim, Germany). According to the Endocrine Society, hyperprolactinemia is defined as prolactin serum levels over 25 µg/L [12]. In addition, patients were subdivided into the following groups according to their prolactin levels: < 7 µg/L, 7–12 µg/L, and > 12 µg/L. We chose this subdivision after the metabolic impact of prolactin and the median prolactin distribution in previous studies: very low prolactin levels < 7 µg/L are deleterious to metabolism, whereas intermediate prolactin levels between 7 and 12 µg/L and high prolactin levels over 12 µg/L seem to be beneficial [19–21, 27, 28].

On the day of blood retrieval, a vaginal ultrasound was also performed with an “Aloka Prosound 6” ultrasound machine and an “UST-9124 Intra Cavity transducer” (frequency range 3.0–7.5 MHz; Wiener Neudorf, Austria). PCOM, the main outcome parameter, was defined as when the number of follicles per ovary exceeded 12 [29]. In addition, the following basic patient characteristics were also included: age at evaluation, body mass index (BMI), and the duration of amenorrhea.

### Statistical analysis

Continuous variables are presented as medians with interquartile ranges (IQR), categorical parameters as numbers and frequencies. The case and the control groups were compared to each other using the analysis of variance (ANOVA, for numerical parameters) and the Fisher’s exact test (for categorical parameters). Factors associated with presence of prolactin levels > 12 µg/L were tested using a multivariable binary logistic regression model. For this model, odds ratios (OR) with their 95% confidence intervals (95% CI) and *p*-values are provided. Using the IBM Statistical Package for Social Science software 26.0 (level of significance: *p* < 0.05).

## Results

**Fig. 1:**
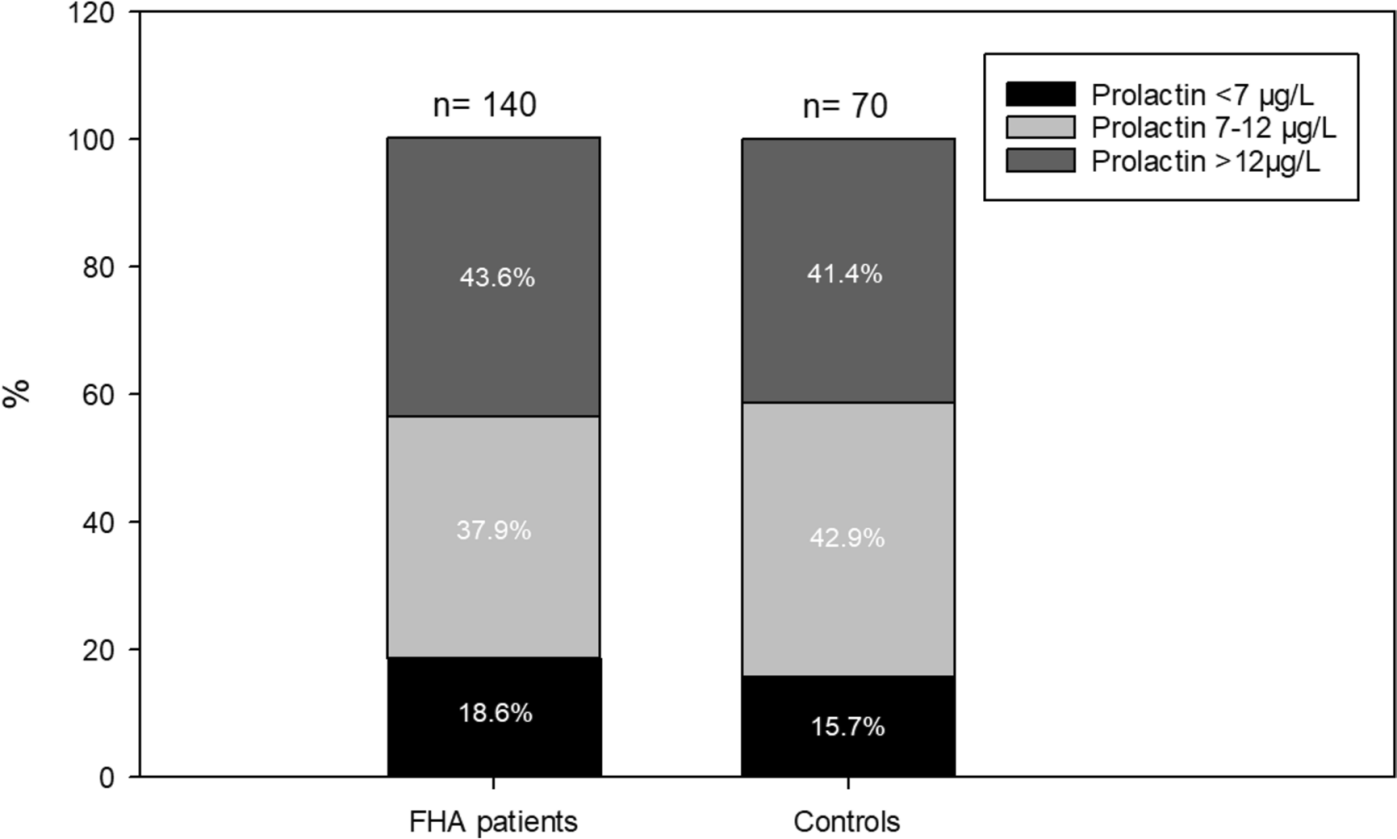
Distribution of prolactin levels in FHA patients and control

In total, 140 women with FHA were included in this study. Basic patient characteristics are provided in Table 1. Notably, the most common cause for FHA was excessive exercise (45.7%), followed by stress (37.9%, multiple mentions possible). The median prolactin level was 11.5 µg/L (IQR 7.5–14.4). Only two women had hyperprolactinemia with prolactin levels > 25 µg/L (1.4%). When FHA patients were divided into 3 different groups according to their prolactin levels according to recent recommendations about the metabolic effects of prolactin [19–21, 27, 28], only 26 women had lower prolactin levels than 7 µg/L (18.6%), 53 women had prolactin levels between 7 and 12 µg/L (37.9%) and the largest group of 61 women had prolactin levels over 12 µg/L (43.6%; Fig. 1) [19–21, 27, 28].

**Table 1:** Basic characteristics of FHA patients and healthy controls

	FHA patients (*n* = 140)	Controls (*n* = 70)	*p*
Age (years)^a^	26 (22; 29)	26 (23; 29)	0.276
BMI (kg/m^2^)^a^	20.0 (18.6; 22.1)	21.5 (19.9; 23.5)	< 0.001
Duration of amenorrhea (months)^a^	13 (7; 24)	–	–
Gravidity ≥ 1^b^	1 (0.7)	10 (14.3)	< 0.001
FHA causes			
Weight loss^b,c^	16 (11.4)	–	–
Underweight^b,c^	12 (8.6)	–	–
Eating disorder^b,c^	19 (13.6)	–	–
Excessive exercise^b,c^	64 (45.7)	–	–
Stress^b,c^	53 (37.9)	–	–
TSH (IU/mL)^a^	1.70 (1.15; 2.33)	1.32 (1.00; 1.75)	0.021
Prolactin (µg/L)^a^	11.5 (7.5; 14.4)	10.7 (8.3; 14.5)	0.605
FSH (mIU/mL)^a^	4.5 (3.2; 6.2)	6.1 (5.0; 7.7)	< 0.001
LH (mIU/mL)^a^	2.6 (1.3; 5.3)	6.0 (3.8; 7.7)	< 0.001
Estradiol (pg/mL)^a^	23 (14; 36)	58 (47; 81)	< 0.001
Testosterone (ng/mL)^a^	0.22 (0.13; 0.30)	0.25 (0.14; 0.30)	0.375
DHEAS (mg/mL)^a^	2.09 (1.42; 2.84)	2.27 (1.88; 2.84)	0.320
SHBG (nmol/L)^a^	72.4 (55.1; 103.5)	77.5 (58.0; 101.3)	0.273
AMH (ng/mL)^a^	3.12 (1.89; 6.06)	3.07 (2.19; 4.12)	0.036
PCOM^b^	61 (43.6)	8 (11.4)	< 0.001

Data are provided as ^a^median (IQR) or ^b^n (%)

^c^Multiple citations for causes of FHA possible

When women with FHA were compared to healthy, ovulating controls, the latter revealed significantly higher FSH (median 6.1 mIU/mL, IQR 5.0–7.7 versus median 4.5, IQR 3.2–6.2; *p* < 0.001), LH (median 6.0 mIU/mL, IQR 3.8–7.7 versus median 2.6 mIU/mL, IQR 1.3–5.3; *p* < 0.001), and estradiol levels (median 58 pg/mL, IQR 47–81 versus median 23 pg/mL, IQR 14–36 *p* < 0.001). Moreover, they had had been pregnant significantly more often (*p* < 0.001). On the other hand, FHA patients had significantly higher TSH levels (median 1.70 IU/mL, IQR 1.15–2.33 versus median 1.32 IU/mL, IQR 1.00–1.75; *p* = 0.021) and revealed PCOM more often (43.6% versus 11.4%, *p* < 0.001). Although the median AMH levels seemed quite similar between the groups, FHA patients showed a larger interquartile range (FHA patients: median 3.12 ng/mL, IQR 1.89–6.06 versus controls: median 3.07 ng/mL, IQR 2.19–4.12; *p* = 0.036). However, there were no differences in median prolactin levels (FHA patients: 11.5 µg/L, IQR 7.5–14.4 versus controls: 10.7 µg/L, IQR 8.3–14.5; *p* = 0.605). Moreover, the prolactin subgroups were distributed equally between patients and controls (*p* = 0.746; Fig. 1). Only two women in the control group (2.9%) revealed prolactin > 25 µg/L, which did not differ from FHA patients (2/140, 1.4%; *p* = 0.602).

In a binary logistic regression model, only patients with FHA were included and parameters associated with prolactin > 12 µg/L were tested (Table 2). The cut-off was chosen in accordance with recent recommendations about metabolic effects of prolactin (see above) and there were too few patients with a prolactin < 7 µg/L to test these in a separate group. In the univariable model, the causes of FHA were of major influence, where stress significantly increased the risk for a higher prolactin (OR 4.321, *p* < 0.001), whereas underweight, eating disorders and excessive exercise decreased the risk (*p* < 0.050). In addition, a higher TSH and the presence of PCOM were found to be positively associated with prolactin levels > 12 µg/L (*p* < 0.050). When all these significant parameters were entered into a multivariable model, only three remained statistically significant: eating disorders and excessive exercise decreased the chance for higher prolactin levels (OR 0.206, *p* = 0.040 and OR 0.280, *p* = 0.031, respectively), whereas a higher TSH was predictive for higher prolactin levels (OR 1.923, *p* = 0.020).

**Table 2:** Factors associated with prolactin > 12 ng/mL

	Prolactin		Univariable models		Multivariable model	
	> 12 ng/mL	≤ 12 ng/mL	OR (95% CI)	*p*	OR (95% CI)	*p*
Age (years)^a^	26 (21; 28)	26 (22; 30)	0.969 (0.905; 1.038)	0.374	–	–
BMI (kg/m^2^)^a^	21.1 (18.8; 22.9)	19.4 (18.6; 20.9)	1.198 (1.029; 1.395)	*0.020*^d^	1.082 (0.898; 1.304)	0.407
Weight loss^b,c^	5 (8.1)	11 (14.1)	1.872 (0.614; 5.705)	0.270	–	–
Underweight^b,c^	1 (1.6)	11 (14.1)	0.100 (0.013; 0.796)	*0.030*^d^	0.188 (0.019; 1.896)	0.156
Eating disorder^b,c^	3 (4.8)	16 (20.5)	0.197 (0.055; 0.711)	*0.013*^d^	0.206 (0.046; 0.932)	*0.040*^d^
Excessive exercise^b,c^	22 (35.5)	42 (53.8)	0.471 (0.238; 0.935)	*0.031*^d^	0.280 (0.088; 0.888)	*0.031*^d^
Stress^b,c^	35 (56.5)	18 (23.1)	4.321 (2.087; 8.945)	< *0.001*^d^	1.250 (0.399; 3.918)	0.702
TSH (IU/mL)^a^	1.88 (1.38; 2.61)	1.50 (1.12; 20.3)	1.834 (1.151; 2.922)	*0.011*^d^	1.923 (1.108; 3.338)	*0.020*^d^
FSH (mIU/mL)^a^	4.6 (3.3; 6.9)	4.5 (2.9; 6.1)	1.055 (0.836; 1.331)	0.653	–	–
LH (mIU/mL)^a^	4.0 (1.3; 7.2)	2.2 (1.2; 4.6)	1.141 (0.956; 1.362)	0.143	–	–
Estradiol (pg/mL)^a^	24 (15; 36)	21 (12; 33)	1.000 (0.988; 1.012)	0.971	–	–
Testosterone (ng/mL)^a^	0.23 (0.12; 0.31)	0.21 (0.13; 0.30)	1.835 (0.094; 35.796)	0.689	–	–
DHEAS (mg/mL)^a^	2.42 (1.50; 3.17)	2.02 (1.40; 2.77)	1.279 (0.962; 1.701)	0.091	–	–
SHBG (nmol/L)^a^	69.1 (55.1; 98.4)	73.4 (55.1; 115.9)	0.993 (0.985; 1.002)	0.145	–	–
AMH (ng/mL)^a^	3.87 (2.24; 6.35)	2.50 (1.63; 4.93)	1.106 (0.990; 1.236)	0.073	–	–
PCOM^b^	34 (54.8)	27 (34.6)	2.294 (1.157; 4.545)	*0.017*^d^	1.788 (0.817; 3.912)	0.146

Results of univariable and a multivariable binary regression models

Data are provided as ^a^median (IQR) or ^b^n (%)

^c^Multiple citations for causes of FHA possible

^d^Italic letters indicate statistical significance

## Discussion

Our retrospective case–control study revealed the following important results: women, who suffer from FHA, showed prolactin levels similar to a population of healthy age-matched controls. Prolactin levels > 25 µg/L were found in 1.4% of FHA patients. Eating disorders, which are associated with decreased calorie intake, as well as excessive exercise were associated with prolactin levels < 12 µg/L, whereas higher TSH levels were linked to higher prolactin levels in women with FHA.

Before discussing the specific results of the present study, the focus should be on the FHA population. As can be seen in Table 1, the FHA patients included in this study, revealed the typical hormonal profile with lower FSH, LH, and estradiol levels as well as a lower BMI than controls. The fact that PCOM can be found frequently in this patient population has already been reported [26, 30, 31]. Given the high rate of FHA women with PCOM and that FHA patients without PCOM are known to have decreased AMH levels, which is likely due to a slower folliculogenesis [14], it is no surprise that the distribution of AMH levels was different between controls and FHA patients with a larger IQR in the latter. TSH levels in FHA women were significantly higher than in the control group. Reduced GnRH pulsatility in women with FHA leads to an impaired hypothalamic–pituitary–thyroid axis, which causes reduced TSH release and, therefore, low-to-normal level of thyrotropin, an increased level of reverse triiodothyronine, and a low level of triiodothyronine [32]. Hence, we find it hard to explain why TSH levels are higher in FHA patients. Lee et al. suggest a possible correlation between PCOM and high TSH levels [33], the high prevalence of PCOM in women with FHA might explain our finding. However, this specific issue needs further investigation. Moreover, it needs to be mentioned that also in the FHA group, the median TSH level was within the normal range (1.70 IU/mL).

Our data demonstrate that women with FHA do not have an increased risk for higher prolactin levels. However, median BMI of women with FHA in the present study was relatively low (20 kg/m^2^) and at the same time eating disorders and excessive exercise were two of the main causes for FHA. In the univariable binary logistic regression models, BMI, excessive exercise and eating disorders were associated with prolactin levels < 12 µg/L (Table 2). This might explain why we could not observe significantly higher prolactin levels in FHA women.

Risk factors for hyperprolactinemia that we know so far include physiological (lactation, pregnancy, sleep, stress), pathological (hypothalamic/pituitary damage, prolactinoma, systemic disorders) or iatrogenic (pharmacological, surgery) causes [12]. There are very few studies investigating the prevalence of hyperprolactinemia with large differences depending on which population group had been observed. The prevalence among the general adult population is estimated to be 0.4% [34].

In the univariable binary logistic regression model, stress and PCOM appear to be linked to higher prolactin levels, which corresponds to prolactin being an important stress hormone [34] and the recent finding that stress-induced FHA is associated with a higher prevalence of PCOM [15]. However, in the multivariable model neither stress nor PCOM were associated with prolactin levels > 12 µg/L. To explain this finding, one might differentiate between acute and chronic stress. Acute stress leads to a short-term adaptive state, whereas chronic stress causes a maladaptive response to a long-lasting condition resulting in inadequate activity of the hypothalamic–pituitary–adrenal axis (HPA-axis), Autonomic Nervous System (ANS), and immune system [35]. The ANS, or more specifically the sympathetic nervous system, responses to stressors with an immediate release of catecholamines. Activation of the HPA-axis starts with the hypothalamus distributing corticotropin-releasing hormone (CRH), amongst others, which leads to the secretion of ACTH in the anterior pituitary, which then leads to the production of glucocorticoids in the adrenal cortex. Glucocorticoid receptors in the medial prefrontal cortex, the hippocampal formation, the paraventricular nucleus and the anterior pituitary are responsible for the negative feedback mechanism of the HPA-axis. Chronic stress lowers ACTH levels due to habituation, but at the same time increases glucocorticoid levels due to stress-induced enhanced capability of the adrenal cortex. Glucocorticoids impede stress-induced prolactin release. This explains, why acute stress causes high serum prolactin levels, whereas chronic stress does not increase or even lower prolactin levels [6, 36]. Considering that only chronic stress plays an important role in the pathogenesis of FHA and the linkage between stress-induced FHA and high prevalence of PCOM, this might explain why we could not observe a significant association between these two parameters and high prolactin levels in the multivariable model [11, 15]. To investigate the pathophysiological interactions between stress, PCOM and prolactin levels in FHA women more thoroughly, further research with a larger sample size might be needed.

Overall, it appears that metabolic causes like excessive exercise, eating disorder and BMI were predominantly influencing prolactin levels in this population of FHA women, even though BMI was only a significant parameter in the univariable model.

In addition, TSH was positively associated with high prolactin levels in the present data set, which corresponds to the finding of previous studies [37–39]. Both, TSH and prolactin, are produced in the anterior lobe of the pituitary gland [40]. Prolactin and TSH release is controlled by triiodothyronine (T3), thyroxine (T4), thyrotropin releasing hormone (TRH), and dopamine, whereby TRH stimulates the release of TSH and prolactin [38]. There is only limited data available regarding the role of TSH in women with FHA. As previously mentioned, higher TSH levels might be explained due to the predominant occurrence of PCOM in the FHA population [33].

## Conclusion

Women with FHA have similar prolactin levels to healthy age-matched individuals. Eating disorders and excessive exercise tend to lower prolactin levels in FHA women, whereas TSH is associated with prolactin levels > 12 µg/L. Moreover, the median TSH level was significantly higher in women with FHA, which could be caused by the high prevalence of PCOM in FHA women [33].

It seems that metabolic effects on prolactin are prevailing in the population of FHA women, even though we could observe a positive association of PCOM and stress with higher prolactin levels in the univariable model. Further investigation with larger sample sizes will be needed to determine the exact influence of stress, PCOM and TSH on prolactin levels in FHA women.

## Data Availability

Data will be provided upon reasonable request.
